# Effects of aerobic and resistance exercise alone or combined on strength and hormone outcomes for people living with HIV. A meta-analysis

**DOI:** 10.1371/journal.pone.0203384

**Published:** 2018-09-04

**Authors:** Camilo Germán Alberto Pérez Chaparro, Philipp Zech, Felipe Schuch, Bernd Wolfarth, Michael Rapp, Andreas Heiβel

**Affiliations:** 1 University Outpatient Clinic–Center for Sports Medicine, Department Sports & Health Sciences, University of Potsdam, Potsdam, Brandenburg, Germany; 2 Social and Preventive Medicine, Department Exercise and Health Sciences, University of Potsdam, Potsdam, Brandenburg, Germany; 3 Post-graduate Program in Health and Human Development, La Salle University, Canoas, RS, Brazil; 4 Department of Sports Sciences, Division of Sports Medicine, Humboldt Universität zu Berlin, Berlin, Germany; UNSW Sydney, AUSTRALIA

## Abstract

**Background:**

Infection with human immunodeficiency virus (HIV) affects muscle mass, altering independent activities of people living with HIV (PLWH). Resistance training alone (RT) or combined with aerobic exercise (AE) is linked to improved muscle mass and strength maintenance in PLWH. These exercise benefits have been the focus of different meta-analyses, although only a limited number of studies have been identified up to the year 2013/4. An up-to-date systematic review and meta-analysis concerning the effect of RT alone or combined with AE on strength parameters and hormones is of high value, since more and recent studies dealing with these types of exercise in PLWH have been published.

**Methods:**

Randomized controlled trials evaluating the effects of RT alone, AE alone or the combination of both (AERT) on PLWH was performed through five web-databases up to December 2017. Risk of bias and study quality was attained using the PEDro scale. Weighted mean difference (WMD) from baseline to post-intervention changes was calculated. The *I*^*2*^ statistics for heterogeneity was calculated.

**Results:**

Thirteen studies reported strength outcomes. Eight studies presented a low risk of bias. The overall change in upper body strength was 19.3 Kg (95% CI: 9.8–28.8, p< 0.001) after AERT and 17.5 Kg (95% CI: 16–19.1, p< 0.001) for RT. Lower body change was 29.4 Kg (95% CI: 18.1–40.8, p< 0.001) after RT and 10.2 Kg (95% CI: 6.7–13.8, p< 0.001) for AERT. Changes were higher after controlling for the risk of bias in upper and lower body strength and for supervised exercise in lower body strength. A significant change towards lower levels of IL-6 was found (-2.4 ng/dl (95% CI: -2.6, -2.1, p< 0.001).

**Conclusion:**

Both resistance training alone and combined with aerobic exercise showed a positive change when studies with low risk of bias and professional supervision were analyzed, improving upper and, more critically, lower body muscle strength. Also, this study found that exercise had a lowering effect on IL-6 levels in PLWH.

## Introduction

According to the Joint United Nations Program on HIV/AIDS, 17 million people living with HIV (PLWH) receive anti-retroviral drug therapy worldwide [1]. This is noteworthy because early use of combined anti-retroviral drug therapy (ART) has been shown to increase life expectancy by ~ 43.1 years [2]. Thus, human immunodeficiency virus (HIV) has changed from an acute and fatal disease into a chronic disease that comprises a greater proportion of persons older than 50 years [3].

Human immunodeficiency virus (HIV) infection affects not only the immune system [4], but also the musculoskeletal system. In particular, PLWH present pre-sarcopenia or sarcopenia [5]. Other associated muscle problems like myalgia are twice as common among PLWH, with or without receiving ART treatment [6]. Low bone mineral density (BMD) is also directly affected by HIV infection, due to the virus and/or use of ART, which stimulates osteoclastic activity and decreases osteoblast activity, affecting BMD in naive ART people [7]. These changes to the musculoskeletal system may be mediated by changes in interleukins, cortisol or testosterone [8–10] and result in a decreased capacity to carry out activities of daily life [11].

Resistance exercise training both alone (RT) and combined with aerobic exercise (AERT) is linked to improved BMD [12], muscle mass and strength maintenance, complemented by weight gain [13–15] and fewer episodes of falls [16] in the general population. These exercise benefits in PLWH have been the focus in different systematic reviews and meta-analyses [17–21]. Gomes Neto et al. [18] showed an improvement (WMD = 25.1 kg, p< 0.001) in knee extensor muscle strength (n = 2 studies) after AE intervention. This finding is in line with those of O´Brien et al.’s [20,21] meta-analysis showing a significant improvement (WMD = 10.5 kg 1-RM, p< 0.001) in knee flexion strength (n = 3 studies) after AERT intervention. However, O´Brien et al.´s [20,21] reported a non-significant improvement in other lower extremity muscle groups through resistance training combined with aerobic exercise. Subgroup analyses were only performed by O-Brien et al. [21] dealing with testosterone supplementation, with a non-significant improvement in knee flexion/extension strength (n = 2 studies). Nevertheless, these results need to be interpreted carefully because of the low number of investigated studies. Also, previous studies [22–24] have suggested that the length of the intervention and supervision by exercise professionals may moderate the benefits of exercise on physical and mental health outcomes. However, none of the previous meta-analyses have investigated whether or not longer interventions and the supervision of exercise modify the effects of exercise on PLWH.

This systematic review and meta-analysis explored the effects of exercise on body strength and hormonally levels in PLWH after resistance alone or combined with aerobic exercise intervention. Likewise, other physiological parameters (i.e. cortisol, testosterone) related to strength and HIV were analyzed. This is of importance in this systematic review, since no meta-analyses have addressed this matter to date. Finally, subgroup analysis for each exercise intervention, upper and lower body muscles, professional supervision of exercise, performing more than 150 minutes of exercise per week, and controlling for active control groups and high-quality studies (PEDro score ≥ 5) were conducted in compliance with the Cochrane meta-analysis standards.

## Methods

This systematic review and meta-analysis was registered in the PROSPERO international prospective register of systematic reviews (CRD42018087004) and performed following the guidelines of the Preferred Reporting Items for Systematic Reviews and Meta-Analyses (PRISMA) [25].

### Eligibility criteria

Randomized controlled trials (RCTs) comparing resistance training alone, aerobic exercise alone or aerobic exercise in combination with resistance training against a non-exercising control group (CG) were considered for inclusion. The studies had to include participants with HIV at any stage of the infection process, older than 18 years, with or without co-morbidities, and investigate strength outcomes (lifted external resistance in kg or lbs) as the primary outcome and hormones (i.e. testosterone) related to the muscular system as secondary outcomes, in response to exercise. Aerobic exercise was defined according to the American College of Sports Medicine (ACSM) as “any activity that: uses large muscle groups, can be maintained continuously, and is rhythmic in nature” [26], and resistance training was outlined as “a form of physical activity that is designed to improve muscular fitness by exercising a muscle or a muscle group against external resistance” [27]. Both exercises had to be performed more than two times per week as described by Gomes Neto et al. [18] rather than three times per week as in O´Brien et al. [21], and for at least four weeks. Studies administering steroid supplementation to the IGs and/or CGs were excluded due to the possibility of an additional effect on muscle strength. Other forms of exercise (e.g. tai chi, qi gong) were not considered because tai chi interventions varied in the tai chi practiced forms [28] and homogeneity among the types of exercise performed by the IGs needed to be achieved.

Studies investigating two exercising groups without any non-exercising control groups were considered to be excluded because an exercising control group could lead to a significant improvement in muscle strength, resulting in a decreased ability of the intervention group (IG) to demonstrate minimal changes. Not necessarily physical activities (placebo-treated, social contact exercise recommendations, counseling, recreational activities) and very light physical activity groups, were considered to be active CGs. Groups following their usual activity and explicitly not exercising were considered to be passive CGs.

### Literature search strategy for study identification

A literature search was performed using five databases (clinicaltrials.gov, PEDro physiotherapy evidence database, PubMed, the Cochrane Central Register of Controlled Trials (CENTRAL) and Web of Science), restricted to English-language studies published up to the end of December 2017. Two reviewers (CP and PZ) individually screened and recorded the relevant citations following the above eligibility criteria and recorded them in a standard data format. After selecting the relevant citations by title, the abstracts were screened. After fulfilling both previous steps, full texts were obtained and evaluated. In case of disagreement, both authors discussed their differences until reaching an agreement. If this was not possible, a third author (AH) was consulted to determine the final decision.

Search parameters and syntax were adapted to each database’s requirements. Text words and Combined Medical Subject Headings (MeSH) terms were related to exercise and physiological parameters. The search strategy is presented in Table 1.

**Table 1:** Systematic search strategy.

Database	Combined MeSH terms and text words
PubMed	(HIV) OR (human immunodeficiency virus) AND (exercise OR exercise therapy OR physical activity OR aerobic exercise OR resistance training) AND (hormone OR testosterone OR cardiovascular OR strength OR fitness OR physiological) AND (randomized controlled trial OR randomized OR clinical trials)
Cochrane library	("HIV" OR "human immunodeficiency virus") AND ("exercise" OR "physical activity" OR "aerobic exercise" OR "resistance training" OR "exercise therapy") AND ("hormone" OR "testosterone" OR "cardiovascular" OR "strength" OR "fitness" OR "physiological" AND "randomized controlled trial")
Clinicaltrials.gov	(HIV infection HIV AND exercise AND physiologic OR muscle) and (HIV AND exercise AND cardiovascular)
PEDro physiotherapy evidence database	(HIV exercise muscle) or (HIV exercise cardiovascular) or (HIV exercise hormones)
Web of Science	(HIV AND exercise)

### Data collection

Data was extracted by both reviewers (CP and PZ) independently, using a standard digital sheet form.

Measuring units were independently converted by the two reviewers (CP and PZ), pounds (lbs) to kilograms (kg) and mmol/l to pg/dl. Outcomes reported as Mean ± standard error or Mean change (post minus pre) ± standard error were converted into mean ± standard deviation.

In case of missing relevant data in the selected studies, the original authors were contacted via email asking for the required missing information. If two weeks passed without an answer from the author, the author was kindly reminded and the co-authors were contacted via email. If the author did not answer our emails, then the study was left out of the quantitative synthesis.

### Risk of bias and quality of included studies

CP and PZ individually assessed the risk of bias and the quality of the included studies using the PEDro scale [25,29]. Every PEDro criterion had to be clearly met and described in the selected study. The PEDro scale consists of eleven criteria in which the first “criterion of eligibility” is marked with a “yes” or “no.” If the study had no eligibility criteria, the study was excluded. The rest of the criteria were marked with a checkmark or a “0.” The possible PEDro score range is from 0 to 10. Discrepancies on the studies’ PEDro score between the two reviewers were resolved by a third author (AH). The results of the quality and risk of bias assessment of the included articles are shown in Table 2 in the Results section.

**Table 2:** PEDro scale, quality assessment of included trials in the systematic review.

Study	EC	I	II	III	IV	V	VI	VII	VIII	IX	X	Total
Agin D. 2001	Y	✓	✓	✓	0	0	0	0	0	✓	✓	5
Bhasin S. 2000	Y	✓	0	✓	0	0	✓	0	✓	✓	✓	6
Dolan S. 2006	Y	✓	0	✓	0	0	0	✓	✓	✓	✓	6
Dudgeon WD. 2012	Y	✓	0	✓	0	0	0	0	0	0	✓	3
Farinatti PT. 2010	Y	✓	0	✓	0	0	✓	✓	✓	✓	✓	7
Fitch K. 2012	Y	✓	0	✓	0	0	✓	0	✓	✓	✓	6
Grinspoon S. 2000	Y	✓	0	0	0	0	0	0	0	✓	✓	3
Lox CL. 1995	Y	✓	0	0	0	0	0	✓	0	✓	✓	4
Lox CL. 1996	Y	✓	0	0	0	0	0	✓	0	✓	✓	4
Mendes EL. 2013	Y	✓	0	0	0	0	0	0	0	✓	✓	3
Pérez-Moreno F. 2007	Y	✓	✓	✓	0	0	✓	0	0	✓	✓	6
Shah KN. 2016	Y	✓	✓	✓	0	0	✓	✓	0	✓	✓	7
Zanetti HR. 2016	Y	✓	0	✓	0	0	0	✓	0	✓	✓	5

EC, eligibility criteria; I: allocated randomization of subjects to groups; II: concealed allocation; III: similarities of groups at baseline; IV: blinding of subjects; V: blinding of researchers/evaluators; VI: blinding of assessors; VII: measure of at least one key outcome obtained from more than 85% of subjects initially allocated to groups; VIII: intention to treat; IX: comparison results between groups; X: measured at least one key outcome at two time points; ✓, criterion is present otherwise; 0, criterion is missing.

Studies with a PEDro score ≥ 5 where categorized as high-quality studies, because blinding might be difficult to achieve and maintain for various reasons and is less frequently reported in non-pharmacological treatment RCTs [30,31]. Moreover, Moseley et al. [32] investigated the number of RCTs available in the PEDro database that satisfied the blinding criteria (subject blinding, therapist blinding or assessor blinding) and found a low prevalence of blinding, with 5% using blinded therapists, 9% blinded subjects and only 34% blinded assessors. For these reasons, the total PEDro score for RCTs involving exercise interventions can be affected and thus a PEDro score lower than six can be attained even if the other criteria are satisfied.

### Statistical analysis

The random effect model [33,34] was used to calculate the weighted mean difference (WMD) between intervention and control group changes from baseline and post-intervention. When the change was not available, the change (mean pre-intervention minus mean post-intervention) and the standard deviation according to the Cochran handbook for systematic reviews of interventions [35] were calculated. Parameters were analyzed for upper and lower body strength as well as hormones. Subgroup analyses were performed for resistance training (RT) alone, aerobic exercise combined with resistance training (AERT), upper and lower body muscles, professional supervision of exercise, performing more than 150 minutes of exercise per week, excluding active CGs, pre ART-era, and high-quality studies (PEDro score ≥ 5) [23]. A p value equal to or less than 0.05 was considered significant. Interpretation of the effect size was done with the most commonly used cut-off defined by Cohen [36], d = .20 small, .50 medium and .80 large.

To test for heterogeneity, the *I*^*2*^ statistics and 95% CI [37] was calculated. Values between 25–50% reflect low, 50%-75% moderate, and values greater than 75% reflect high heterogeneity [37]. To explore the heterogeneity, this meta-analysis looked for differences across subgroups by calculating the Chi square (Χ^2^). Publication bias was assessed by the egger´s test [38]. All analyses were performed using Review Manager Version 5.3 [39].

## Results

### Search description of selected studies

**Fig 1:**
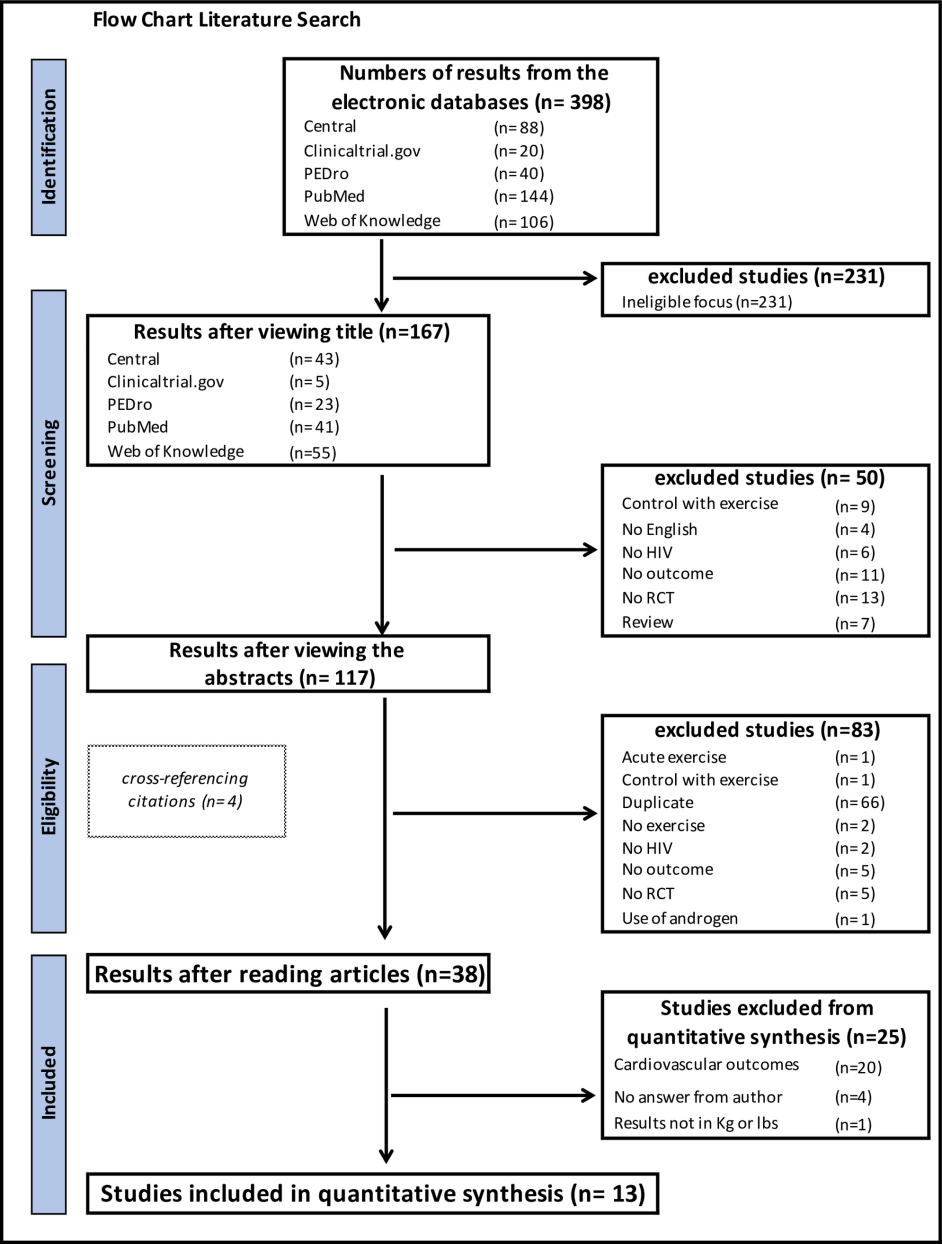
Search and selection of studies from the systematic review according to PRISMA. Physiotherapy Evidence Database (PEDro), Preferred Reporting Items for Systematic Reviews and Meta-Analyses (PRISMA).

A total of 398 citations through the search criteria from the databases described in the Methods section where retrieved. After screening the titles, 231 citations were excluded due to ineligible focus. Of the remaining 167 studies, 50 were excluded for the following reasons: exercise was performed by the control group (*n* = 9), the language was other than English (*n* = 4), the intervention or control groups were partially or totally integrated with HIV seronegative participants (*n* = 6), the searched outcomes were not addressed (*n* = 11), or the citations referred to non- RCTs (*n* = 13) or reviews (*n* = 7). The remaining 117 citations were screened before full texts were acquired; 66 citations were duplicates. 51 studies’ full texts were read and 17 studies had to be excluded: in one study, the intervention groups performed only one bout of exercise [40], one study’s control group performed exercise [41], two studies did not investigate exercise [42,43], two studies’ intervention or control groups were partially or totally integrated with HIV seronegative participants [44,45], five studies did not investigate the desired outcomes [46–50], five were not RCTs [51–55] and one study administered nandrolone [56]. In total, 34 studies from the systematic search and four studies added by cross-referencing citations met the eligibility criteria and were considered relevant for inclusion in the meta-analysis. In total, 13 studies reported strength outcomes and were included in the quantitative analysis. Of these, four studies [57–60] reported strength and hormone outcomes (See Fig 1).

### Characteristics of studies excluded from quantitative synthesis

Following our search strategy, five studies [61–65] were excluded due to incomplete data or because the requested data was not available. See excluded studies in S1 Table, Information on excluded and included studies.

### Description of the studies included in the meta-analysis

Of the 13 included studies, eight studies [57,60,66–71] were classified as high-quality studies (see Table 2), four studies investigated RT [57,60,66,72] and eight studies AERT [58,59,67–71,73]. The studies of Lox et al. [74] and [72] shared the same two intervention groups and results: one group performed AE alone and the other RT alone, with the exact same number of control group participants in both studies. Thus, for the purpose of analysis we decided to treat Lox et al. [74] as an AE-only intervention and Lox et al. [72] as an RT-only intervention. Passive CGs were identified in six studies [58,60,67,68,71,73] and one with no lifestyle modification [71]. Seven studies had active CGs, three studies CGs performed very low-intensity physical activity like walking or stretching [70,72,74], one study [66] applied protein supplementation 1 g·kg^-1^·day^-1^ to the CG, and three studies [57,59,69] administered testosterone placebo injections to the CGs. Characteristics of all the included studies can be seen in S1 Table.

### Characteristics of studies included in the meta-analysis

The number of participants included in the 13 studies were for the intervention group n = 249 at baseline and n = 246 post-intervention, whereas for the control group n = 1216 at baseline and n = 210 at the end of the study. Eight studies had a mean dropout rate of 10.7 ± 12.1%. Two studies reported no dropouts [60,68] and three studies [72–74] only reported participants who completed the study. The mean age for the control group was 42 ± 5.7 years and for the intervention group 42.9 ± 5.3 years. Five studies [60,66,67,69,71] included 42 women in the control group and 41 women in the intervention group. Seven studies recruited only male subjects [57–59,68,70,72,74]. Only the study by Mendes et al. [73] did not report the age and gender of the participants. The average baseline CD4 cell count was reported in 12 studies [57–60,66–72,74]. For the CGs was 432.2 ± 147.9 cells μl^-1^ and for the IGs 431.5 ± 167 cells μl^-1^. Five studies included participants with health-related conditions aside from HIV [57,67,69–71]. Two studies included participants with AIDS wasting syndrome [57,59], one study lipodystrophy [67], one study low testosterone levels [57], two studies metabolic diseases [69,71] and one study included participants with functional limitations [71].

Nine studies reported strength outcomes without hormone outcomes [66–74]. Two studies [57,59] reported strength outcomes and testosterone. Three studies [57–59] reported strength outcomes and free testosterone. The study by Dudgeon et al. [58] reported strength outcomes, cortisol and insulin-like growth factor 1 (IGF-1). Two studies [58,60] reported strength outcomes and pro-inflammatory interleukins (IL-6 and IL-1β). The study by Fitch et al. [69] reported strength outcomes and c-reactive protein (CRP). Long-term effects (> 4 months) of exercise were identified on three [66,69,73] of the 13 included studies.

The 1-RM test used to measure muscle strength was reported in six studies [57,60,66,67,69,73] and the peak isometric force test was used in three studies [59,72,74]. Characteristics of all the included studies can be seen in S1 Table.

### Risk of bias

According to the risk of bias analysis, eight studies scored ≥ 5 in the PEDro scale, specifying a low risk of bias. Five studies presented a high risk of bias with a PEDro score < 5. See Table 2.

### Changes on body strength in PLWH

All types of repetition maximum (1-RM, 3-RM, 6-RM and 12-RM) were included. No details on the type of machine used to perform the RM test was mention in the included studies. See S1 Table.

**Fig 2:**
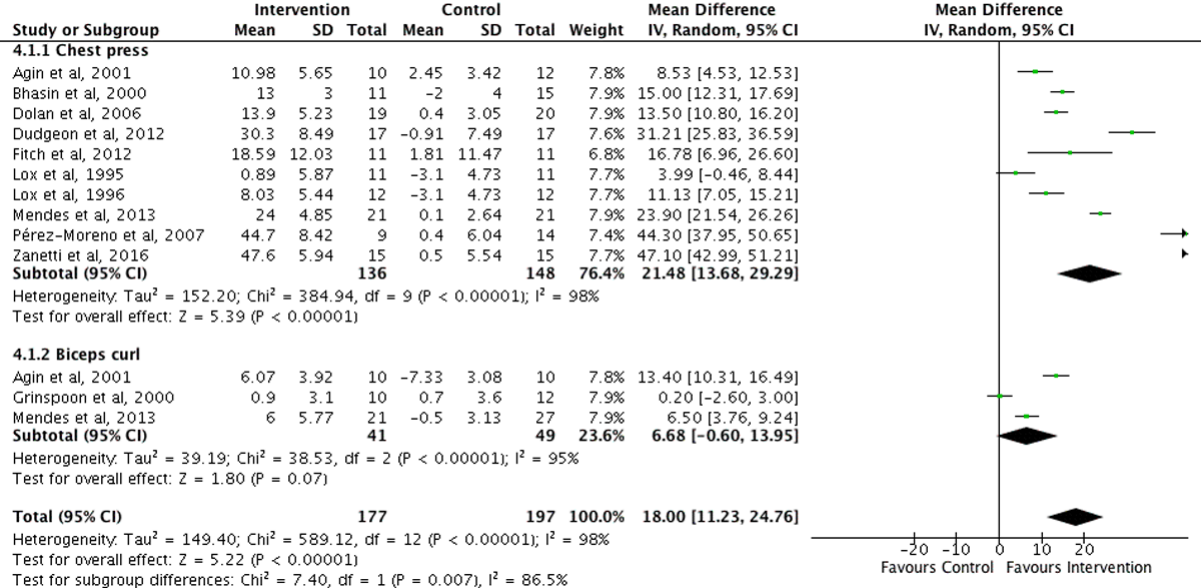
Upper body strength changes after exercise in PLWH. Standard deviation (SD), total (*n* participants), 95% confidence interval (95% CI), Z-score (Z), significance (*p*).

**Fig 3:**
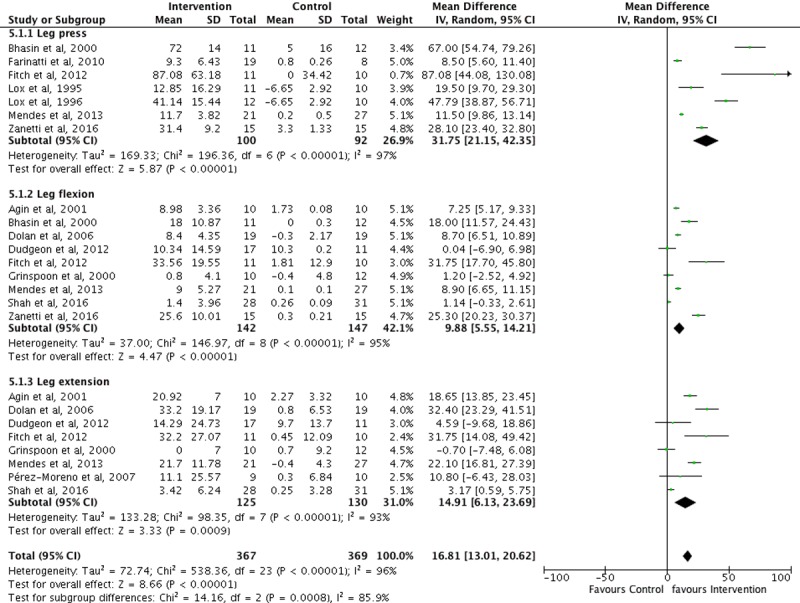
Lower body strength changes after exercise in PLWH. Standard deviation (SD), total (*n* participants), 95% confidence interval (95% CI), Z-score (Z), significance (*p*).

Two overall meta-analyses (see Figs 2 and 3) and 34 subgroup analyses were performed. The overall change after intervention on upper body strength in PLWH from baseline was 18 kg (95% CI: 11.2–24.8, p< 0.001) favoring the IG. Lower body strength also increased by 16.8 kg (95% CI: 13–20.6, p< 0.001) favoring the IG. Sub-analysis revealed a significant increase on lifted weight for each muscle group, favoring the IG. After long-term exercise, IG upper body strength showed a significant change 13.7 kg (95% CI: 6–21.5, p < 0.001). This was also true for IG lower-body strength with a mean change of 16 kg (95% CI: 11.6–20.4, p < 0.001), but significant changes were only for leg flexion and extension long-term exercise muscle groups (See Table 3).

**Table 3:** Body strength changes from baseline in PLWH.

Category	*n* trials (*n* participants)	Change (kg)		Overall effect	Heterogeneity
		WMD	95% CI	Z (*p*)	I^2^
**Upper body strength**					
Chest press	10 (284)	21.5	13.7–29.2	5.4 (< 0.00001)	98%
Biceps curl	4 (90)	6.7	-0.6–13.9	1.8 (0.07)	95%
*Long-term exercise*					
Chest press	3 (86)	16.4	4.8–28.1	2.8 (0.0006)	95%
Biceps curl	2 (68)	9.9	3.1–16.7	2.9 (0.004)	91%
*High-quality studies*					
Chest press	6 (162)	24.2	11.9–36.5	3.8 (< 0.0001)	98%
*AERT*					
Chest press	5 (160)	25.9	16.8–35.1	5.5 (< 0.00001)	96%
Biceps curl	2 (70)	3.4	-2.8–9.5	1.1 (0.29)	90%
*RT*					
Chest press	4 (102)	18.9	17.2–20.7	21 (< 0.00001)	99%
*Professional supervision*					
Chest press	7 (190)	17.9	10.5–25.3	4.8 (< 0.00001)	96%
*Post-ART era*					
Chest press	8 (238)	25	16.3–33.7	5.6 (< 0.00001)	98%
Biceps curl	4 (90)	6.7	-0.6–13.9	1.8 (0.07)	95%
*Only passive controls*					
Chest press	4 (145)	28.8	15.8–41.9	4.3 (< 0.00001)	98%
**Lower body strength**					
Leg press	7 (192)	31.7	21.1–42.3	5.9 (< 0.00001)	97%
Leg flexion	9 (289)	9.9	5.5–14.2	4.5 (< 0.00001)	95%
Leg extension	8 (255)	14.9	6.1–23.7	3.3 (0.0002)	93%
*Long-term exercise*					
Leg press	2 (69)	46.1	-27.7–119.9	1.2 (0.22)	92%
Leg flexion	3 (89)	10.3	5.5–15	4.2 (< 0.0001)	83%
Leg extension	3 (89)	20.9	16.7–25	9.9 (< 0.00001)	19%
*High-quality studies*					
Leg press	4 (101)	41.1	19.1–63.1	3.6 (0.0003)	98%
Leg flexion	6 (191)	13.5	7.4–19.7	4.3 (<0.0001)	96%
Leg extension	5 (157)	18.7	6.3–31.2	2.9 (0.003)	94%
*AERT*					
Leg press	3 (96)	11.6	5.4–17.8	3.7 (0.0002)	87%
Leg flexion	6 (216)	6.2	1.7–10.7	2.7 (0.007)	93%
Leg extension	7 (235)	14.4	4.2–24.5	2.8 (<0.00001)	93%
*RT*					
Leg press	3 (75)	47	25.2–68.9	4.2 (< 0.0001)	95%
Leg flexion	3 (73)	16.7	4.4–29	2.7 (0.008)	96%
*Professional supervision*					
Leg press	4 (93)	35.3	9–61.7	2.6 (0.009)	98%
Leg flexion	4 (109)	8.5	4.5–12.4	4.2 (< 0.0001)	80%
Leg extension	4 (105)	18	7.5–28.6	3.3 (< 0.0008)	77%
*Post-ART era*					
Leg press	6 (149)	29.9	18.5–41.4	5.1 (< 0.00001)	97%
Leg flexion	9 (289)	9.9	5.5–14.2	4.5 (< 0.00001)	95%
Leg extension	8 (255)	14.9	6.1–23.7	3.3 (0.0009)	93%
*Only passive controls*					
Leg press	3 (105)	15.8	7.4–24	3.7 (0.0002)	96%
Leg flexion	5 (203)	8.8	2.6–15	2.8 (0.006)	96%
Leg extension	4 (173)	15.7	1.1–30.3	2.1 (0.03)	96%

Subgroup analyses are presented in random effect model. Panel includes *n* trials (*n* participants), weighted mean difference (WMD), 95% confidence interval (95% CI), Z-score (Z) and significance (*p*) of exercise vs. control conditions. Statistical heterogeneity (I^2^). AERT = aerobic exercise combined with resistance training, RT = resistance training, ART = antiretroviral therapy. Not able to calculate (NA)

Eight studies had a PEDro score ≥ 5. This sub-analysis revealed a greater change in IG upper body strength of 22.6 kg (95% CI: 12.5–32.7, p< 0.001) and 20.1 kg (95% CI: 14.7–25.4, p< 0.001) for lower body strength, compared to the two main meta-analyses.

Aerobic exercise combined with resistance training prompted a greater change in IG upper body strength of 19.3 kg (95% CI: 9.8–28.8, p< 0.001) compared to RT alone, where the change was only 17.5 kg (95% CI: 16–19.1, p< 0.001). In contrast, RT alone provoked a higher change in IG lifted lower body weight by 29.4 kg (95% CI: 18.1–40.8, p< 0.001), while the change in aerobic exercise combined with resistance training was only 10.2 kg (95% CI: 6.7–13.8, p< 0.001). Only the study of Lox et al. (95) reported strength changes from aerobic exercise alone, with a difference after intervention in the IG of 0.9 kg, with a small effect size of 0.02 for the upper body strength and 12.8 kg with a moderate effect size of 0.59 for the lower body strength.

Professional supervision was reported for upper body strength in seven studies. Only the chest press muscle group was reported in these studies, with a significant change in the IG of 17.9 kg (95% CI: 10.5–25.3, p < 0.001).

Eight studies reported a change in lower body strength under professional supervision in the IG of 19.5 kg (95% CI: 13.4–25.7, p< 0.001), which was higher than the main meta-analysis. See Table 3.

After excluding studies pre-ART era, performed or published before or in the year 1996 [75], a significant change in the IG’s upper body strength (19.9 kg [95% CI: 12.3–27.5], p < 0.001) and lower body strength (15.1 kg [95% CI: 11.4–18.8], p < 0.001) was found, where the result for IG upper body strength was higher than that of the main meta-analysis.

Analysis of IG body strength, after excluding studies using active control groups, found a significant change for upper body strength (24.3 kg [95% CI: 12–36.7], p < 0.001) and lower body strength (12.8 kg [95% CI: 8.8–16.8], p < 0.001), although the results from the main meta-analyses were higher for lower body strength. Upper and lower body strength heterogeneity was high (> 75%) for the two main meta-analyses and all sub-analyses. See Table 3.

### Hormone changes in PLWH

After screening the included studies, this meta-analysis found that three studies reported inflammatory markers (IL-6,IL-1β, CRP) and given their relation to the muscular system they were included in the following section.

Out of the three studies that reported hormone outcomes in PLWH, two studies [58,59] had a high risk for bias with a PEDro score ≤ 5. Two studies reported testosterone [57,59], three studies free testosterone [57–59] and two studies interleukin-6 and interleukin-1β [58,60]. All studies reported that samples were taken in a fasting state at the same time of day and in a rested state. For characteristics of the studies, please refer to S1 Table.

Four overall analyses and four sub-meta-analyses were performed. There were no significant differences between IG and CG from baseline to post-intervention in PLWH for total testosterone, and the range of change was widely spread across the main meta-analyses. Likewise, free testosterone and IL-1β also presented a non-significant change between IGs and CGs after intervention (see Table 4).

**Table 4:** Hormone changes from baseline in PLWH.

Category	*n* trials (*n* participants)	Change		Overall effect	Heterogeneity
		WMD	95% CI	Z (*p*)	I^2^
Testosterone (ng/dl)	2 (45)	40.8	-20.8–102.5	1.3 (0.19)	0%
Free testosterone (pg/dl)	3 (73)	-3	-8–2.1	1.1 (0.25)	0%
AERT	2 (50)	-3.4	-8.8–2	1.2 (0.21)	0%
Professional supervision	2 (51)	0	-11.4–11.5	0 (0.99)	0%
IL-6 (pg/ml)	2 (58)	-2.4	-2.6, -2.1	18.6 (< 0.00001)	0%
Only passive controls	2 (58)	-2.4	-2.6, -2.1	18.6 (< 0.00001)	0%
IL-1β (pg/ml)	2 (58)	-0.2	-3–2.6	0.1 (0.88)	71%
Only passive controls	2 (58)	-0.2	-3–2.6	0.1 (0.88)	71%

Subgroup analyses are presented in random effect model. Panel includes *n* trials (*n* participants), Weighted mean difference (WMD), 95% confidence interval (95% CI), Z-score (Z) and significance (*p*) of exercise vs. control conditions. Statistical heterogeneity (I^2^). AERT = aerobic exercise combined with resistance training, IL = interleukin. Not able to calculate (NA)

A significant change towards lower levels of IL-6 in the IG was found, with a WMD reduction of -2.4 ng/dl (95% CI: -2.6 to -2.1, p< 0.001) (see Table 4).

Only the study by Dudgeon et al. [58] reported changes in cortisol and insulin-like growth factor 1 (IGF-1) for PLWH. After exercise intervention, lower levels of cortisol upon waking (p< 0.05) and one hour after waking (p = 0.07) were found in the IG. Overall cortisol levels prior to exercise decreased in the IG after the aerobic exercise combined with resistance training (AERT) intervention by 1.8 μg/ml (55.6 ± 24.3 to 61.2 ± 37.1, p = 0.3), while in the CG cortisol levels increased by 12.2 μg/ml (45.5 ± 22.8 to 57.7 ± 27.8, p = 0.1. IGF-1 had a non-significant increase in IGs and CGs at the end of the study, IG (pre: 128 ± 42 ng/ml; post: 132 ± 43.2 ng/m; p> 0.05) CG (pre: 118 ± 33.8 ng/ml; post: 137 ± 55.3 ng/ml; p> 0.05). C-reactive protein was reported in the study by Fitch et al. [69], where AERT exercise significantly decreased the levels of this inflammatory marker in the IG compared to the CG (−1.6 ± 0.7 vs. 0.1 ± 0.4 mg/L, p = 0.05).

## Differences across subgroups

The heterogeneity was explored by looking for differences across subgroups with and without comorbidities, where studies investigating biceps curl (Χ^2^ = 6.8, df = 1, p < 0.05), leg press (Χ^2^ = 36.7, df = 1, p < 0.001) and leg extension (Χ^2^ = 21.4, df = 1, p < 0.001) differed. A low heterogeneity was evident for leg press (0%) and moderate for leg extension (62%) in the group without comorbidities.

Biceps curl (Χ^2^ = 38.5, df = 1, p < 0.001), leg press (Χ^2^ = 17,4, df = 2, p < 0.001) and leg extension (Χ^2^ = 10.5, df = 2, p < 0.001) differed between studies including only women or men and mixed groups. The heterogeneity was low in the female group for leg flexion (0%) and moderate for CP (75%). The heterogeneity was low in the male group for leg extension (0%). This last subgroup analysis revealed a greater change in IG lower body strength of 17.8 kg (95% CI: 13.1–22.4, p < 0.001).

Regarding the type of training, chest press (Χ^2^ = 17.4, df = 2, p < 0.001), biceps curl (Χ^2^ = 8.13, df = 1, p = 0.004) and leg press (Χ^2^ = 10.1 df = 2, p < 0.001) were also different among the AE, RT and AERT groups. The heterogeneity did not change and was high for type of training ranging from 93% to 96%.

Differences between studies reporting strength intensities and not reporting strength intensities investigating chest press (Χ^2^ = 21, df = 1, p <0.001) and leg press (Χ^2^ = 9.1, df = 1, p = 0.003) were found. A low heterogeneity was evident for studies reporting no strength intensity for leg extension (0%).

Differences between weekly volume sessions (the number of RT sets, times the number of training session per week: < 5, 5–9, ≥ 10 times per week) investigating chest press (Χ^2^ = 18.4, df = 2, p <0.001) and leg flexion (Χ^2^ = 8.5, df = 2, p = 0.01) were found. The heterogeneity did not change and was high in the weekly volume sessions subgroup analysis ranging from 79% to 99%).

Differences between the minutes of physical activity per week in total investigating chest press (Χ^2^ = 15.1, df = 2, p <0.001), leg press (Χ^2^ = 6.2, df = 1, p = 0.01), and leg extension (Χ^2^ = 7, df = 2, p = 0.03) were found. A moderate heterogeneity of the studies investigating chest press for the subgroup performing 180 min/week of exercise was evident for leg extension (64%). A moderate heterogeneity for the subgroup performing 240 to 270 min/week was found for leg press (68%) and leg extension (67%).

The strength tests used in the studies indicated that the chest press (Χ^2^ = 17.4, df = 2, p < 0.001), biceps curl (Χ^2^ = 6.8, df = 1, p = 0.009), leg press (Χ^2^ = 13, df = 2, p = 0.002), leg flexion (Χ^2^ = 128 df = 2, p < 0.001) and leg extension (Χ^2^ = 37.6, df = 1, p < 0.001) differed among the studies. The heterogeneity was high for chest press (81%) when strength was assessed by the peak force test and moderate for leg extension when strength was assessed by the 1-RM method. Heterogeneity was low for leg flexion (0%) and leg extension (0%) in the group that used other methods to asses muscle strength (3-RM, 6-RM, 12-RM or by a hand-held dynamometer) (see Table 5).

**Table 5:** Sub-group differences.

Category	*n* trials (*n* participants)	Change (kg)		Overall effect	Heterogeneity	Difference	
		WMD	95% CI	Z (*p*)	I^2^	χ^2^	(*p*)
Comorbidities							
CP with	4 (110)	22.2	11.7–32.8	4.12 (< 0.0001)	96%	0.02	0.88
CP without	6 (174)	21	9–32.9	3.4 (0.0006)	98%		
BC with	1 (22)	0.2	-2.6–3	0.14 (0.89)	NA	6.8	0.009
BC without	2 (90)	9.9	3.1–16.7	2.9 (0.004)	91%		
LP with	2 (44)	68.5	56.7–80.3	11.39 (< 0.00001)	0%	36.7	<0.00001
LP without	5 (148)	22.3	13.1–31.5	4.77 (< 0.00001)	96%		
LF with	5 (163)	9.4	3.4–15.4	3.1 (0.002)	94%	0.06	0.81
LF without	4 (126)	10.5	3.9–17.1	3.11 (0.002)	94%		
LE with	5 (159)	5.2	2.9–7.5	4.4 (<0.00001)	92%	44.5	<0.00001
LE without	3 (96)	19.3	15.8–22.7	11 (<0.00001)	62%		
Gender							
CP women	2 (61)	11.2	6.4–16.1	4.5 (<0.00001)	75%	4	0.14
CP mixed	2 (52)	32.3	2.6–62	2.1 (0.03)	97%		
CP males	5 (129)	20.9	9.6–32.3	3.6 (0.0003)	97%		
BC women	1 (20)	13.4	10.3–16.5	8 (< 0.00001)	NA	38.5	<0.00001
BC males	1 (22)	0.2	-2.6–3	0.1 (0.89)	NA		
LP mixed	2 (31)	53.6	-3.7–110.8	4.4 (<0.0001)	86%	0.3	0.57
LP male	4 (93)	35.4	9–61.7	2.6 (0.009)	98		
LF women	2 (58)	7.9	6.4–9.4	10.3 (<0.00001)	0%	1.2	0.55
LF mixed	3 (110)	18.7	-1.5–39	1.8 (0.07)	98%		
LF male	3 (73)	6.3	-4.3–16.9	1.2 (0.25)	91%		
LE women	2 (58)	25	11.5–38.4	3.6 (0.0003)	85%	17.9	0.0001
LE mixed	2 (80)	16.1	-11.8–43.9	1.1 (0.26)	90%		
LE male	3 (69)	1.4	-4.3–7.2	0.5 (0.62)	0%		
Type of training							
CP AE	1 (22)	4	-0.5–8.4	1.8 (0.08)	NA	20	<0.0001
CP AERT	5 (160)	25.9	16.8–35.1	5.5 (<0.00001)	96%		
CP RT	4 (102)	20.4	4.5–36.3	2.5 (0.01)	99%		
BC AERT	2 (70)	3.4	-2.8–9.5	1.1 (0.29)	90%	8.1	0.004
BC RT	1 (20)	13.4	10.3–16.5	8.5 (<0.00001)	NA		
LP AE	1 (21)	19.5	9.7–29.3	3.9 (<0.0001)	NA	10.1	0.006
LP AERT	3 (96)	11.6	5.4–17.8	3.7 (0.0002)	87%		
LP RT	3 (75)	47	25.2–68.9	4.2 (<0.0001)	95%		
LF AERT	6 (216)	6.2	1.7–10.7	2.7 (0.007)	93%	2.5	0.12
LF RT	3 (73)	16.7	4.4–29	2.7 (0.008)	96%		
LE AERT	7 (235)	14.4	4.2. 24.5	2.8 (0.006)	93%	0.6	0.4
LE RT	1 (20)	18.7	13.8–23.4	7.6 (<0.00001)	NA		
Strength training intensity							
CP 50–90%	6 (175)	14.8	9.7–19.8	5.7 (<0.00001)	92%	21	<0.00001
CP unknow	3 (87)	40.9	30.9–50.9	5.7 (<0.00001)	91%		
LP 50–90%	5 (141)	35.7	22.3–49.2	5.2 (<0.00001)	97%	1.1	0.29
LP unknow	1 (30)	28.l	23.4–32.8	11.7 (<0.00001)	NA		
LF 50–90%	6 (172)	9.4	5.9–12.9	5.2 (p<0.00001)	86%	0.1	0.76
LF unknow	2 (89)	13.1	-10.6–15.5	1.1 (0.28)	99%		
LE 50–90%	5 (149)	19.9	9.3–30.4	3.7 (0.0002)	91%	9.1	0.003
LE unknow	2 (87)	3.2	0.7–5.8	2.5 (0.01)	0%		
Weekly volume sessions							
CP < 5	1 (34)	31.2	25.8–36.6	11.3 (<0.00001)	NA	18.4	0.0001
CP 5–9	6 (166)	20.6	19.2–22	28.9 (<0.00001)	98%		
CP > 10	2 (62)	18.2	15.7–20.7	14.4 (<0.00001)	99%		
LF < 5	1 (28)	0.04	-6.9–7	0.01 (0.99)	NA	8.5	0.01
LF 5–9	6 (162)	13.7	7.6–19.7	4.4 (<0.00001)	94%		
LF > 10	1 (38)	8.7	6.7–15.4	5 (<0.00001)	NA		
LE < 5	1 (28)	4.6	-9.7–18.9	0.6 (0.53)	NA	2.6	0.28
LE 5–9	4 (101)	16.7	5.4–28	2.9 (0.004)	91%		
LE > 10	2 (57)	22.9	1.9–43.9	2.1 (0.03)	79%		
Minutes week							
CP 180	4 (90)	9	4.9–13.2	4.3 (<0.0001)	64%	15.2	0.0005
CP 240–270	3 (109)	32.8	21.4–44.3	5.6 (<0.00001)	95%		
CP 360	1 (39)	13.5	10.8–16.2	9.8 (<0.00001)	NA		
BC 180	2 (42)	6.1	4.1–8.2	5.8 (<0.00001)	97%	0.04	0.84
BC 240	1 (48)	6.5	3.8–9.2	4.7 (<0.00001)	NA		
LP 180	3 (64)	44.5	17.6–71.4	3.2 (0.001)	91%	6.2	0.01
LP 240–270	2 (75)	10.2	7.3–13.1	6.9 (<0.00001)	68%		
LF 180	3 (63)	9.5	1.3–17.7	2.2 (0.02)	90%	0.7	0.71
LF 240	2 (78)	5.1	-3.5–13.7	1.2 (0.24)	82%		
LF 360	1 (38)	8.7	6.5–10.9	7.8 (<0.00001)	NA		
LE 180	3 (63)	15.1	-1.1–31.5	1.8 (0.07)	92%	7	0.03
LE 240–270	3 (95)	14.1	2.1–26.2	2.3 (0.02)	67%		
LE 360	1 (38)	32.4	23.3–41.5	7 (<0.00001)	NA		
Strength test							
CP 1-RM	6 (181)	20.9	11.3–30.4	4.3 (<0.0001)	98%	17.4	0.0002
CP peak force	2 (46)	7.6	0.6–14.6	2.1 (0.03)	81%		
CP other	2 (57)	37.6	24.8–50.5	5.7 (<0.00001)	89		
BC 1-RM	2 (68)	9.9	3.1–16.7	2.9 (0.004)	91%	6.8	0.009
BC peak force	1 (22)	0.2	-2.6–3	0.1 (0.89)	NA		
LP 1-RM	4 (122)	41.1	21.1–61.1	4 (<0.0001)	98%	13	0.002
LP peak force	2 (43)	33.7	6–61.4	2.4 (0.02)	94%		
LP other	1 (27)	8.5	5.6–11.4	5.7 (<0.00001)	NA		
LF 1-RM	6 (180)	14.4	9.6–19.1	6 (<0.00001)	92%	28	<0.00001
LF peak force	1 (22)	1.2	-2.5–4.9	0.6 (0.53)	NA		
LF other	2 (87)	1.1	-0.3–2.5	1.5 (0.14)	0%		
LE 1-RM	4 (127)	24.1	18–30.3	7.7 (<0.00001)	63%	41.3	<0.00001
LE peak force	1 (22)	-0.7	-7.5–6.1	0.2 (0.84)	NA		
LE other	3 (106)	3.4	0.9–5.9	2.6 (0.009)	0%		

Subgroup analyses are presented in random effect model. Panel includes *n* trials (*n* participants), Weighted mean difference (WMD), 95% confidence interval (95% CI), Z-score (Z) and significance (*p*) of exercise vs. control conditions. Statistical heterogeneity (I^2^). Subgroup differences are tested by the Chi-square (χ^2^) and significance (*p*). Not able to calculate (NA)

## Discussion

The main results of this meta-analysis suggest that aerobic exercise combined with resistance training or resistance training alone has a positive effect on muscle strength and levels of IL-6 in PLWH.

New in this meta-analysis is the inclusion of 13 studies reporting changes in strength, testosterone and inflammatory markers in PLWH. Moreover, separate analyses of the main outcomes for RT alone and AERT, studies with a low risk of bias, professional supervision and the exclusion of active control groups were performed.

### Muscle strength

As previously stressed by Gomes-Neto et al. [18], aerobic exercise and resistance training programs have limited benefit on muscle strength in healthy people. Nonetheless, two previous meta-analyses have reported positive changes after AERT intervention in muscle strength in PLWH [18,21]. However, these changes were restricted only to chest press, biceps curl, and leg extensors, with no changes to the leg flexors [21]. Our results indicated that both RT alone and AERT exercise has a significant positive effect on increasing upper and lower body muscle strength in PLWH. This study highlighted that the results were significantly higher after excluding studies with a high risk of bias over upper and lower strength.

Of importance here is that after different sub-meta-analyses were performed, lower strength increased more after RT alone or if the exercise was supervised by an exercise professional (exercise scientist, physiotherapist or certified trainer [23]). Upper strength increased more after AERT intervention, excluding active CGs and in studies carried out during the ART era.

Of special importance is the significant positive change in the leg press, leg flexors and leg extensors (see Table 3). The data contrasts with that of O´Brien et al. [21] who reported only a trend toward increasing leg flexor muscle strength and a non-significant improvement on other lower extremity muscle groups in LPWH. This difference in results might be due to the increased number of included studies in the meta-analyses for lower body strength in comparison to O´Brien et al. [21]. In detail, for leg press six further studies [57,60,68,72–74], for leg flexion six further studies [57,58,60,66,71,73] and for leg extension five further studies [58,66,70,71,73] were included. It is important to remark that in the study of Dudgeon et al. [58], the intervention group exercised less than three times per week, an exclusion criterion in the study by O’Brien et al. [21]. See S1 Table.

This meta-analysis tried to have a more homogeneous IG by grouping the IGs according to the amount of time (more or less than 150 minutes per week) dedicated to exercise. Due to complex differences in the types of training (progressive resistance training, continuous resistance training, AERT), the use of free weights, color-coded therapeutic bands or multi-training exercise machines, alongside great differences in exercise intensity (refer to S1 Table, Information on excluded and included studies), no differences in strength compared to the main results were found.

The clinical relevance of muscle strength increases in PLWH has not yet been established. Gomes-Neto et al. [18] discussed how an increase of 40% in muscle strength likely represents a clinically meaningful strength gain. On the other hand, for O´Brien et al. [21] a clinically important change was an improvement of 2 kg for upper body and 5 kg for lower body strength. Nevertheless, no statement can be made, about the clinically relevant change due to the different possible biases in our study (i.e. types of training, exercise intensity, strength testing) and because our focus was not the standard error of measurement (SEM) and the small real difference (SRD) values of the upper and lower body strength tests.

The high degree of heterogeneity (*I*^2^> 75%) found among the meta-analyses might be the result of differences in the associated comorbidities (i.e. insulin resistance, HCV and altered testosterone levels), gender (only women were evaluated), and strength testing procedures (1-RM test, 3RM, 6-RM, 12-RM or peak isometric force). Furthermore, the number of participants in the included studies as well as the number of studies included in each of the meta-analyses can contribute to this high heterogeneity. Thus, caution is advised when interpreting the results.

### Hormones

Testosterone levels are associated with muscle strength and physical performance in healthy men [76]. This meta-analysis found no significant changes in testosterone and free testosterone levels after the exercise interventions (see Table 4), perhaps due to differences in the testing methods, the number of included studies and, more importantly, because this secondary outcome was extracted from studies mainly reporting changes in muscle strength.

High levels of IL-6 are related to T-cell failure [77], a greater risk of losing 40% of muscle strength [76] in the aging population, and a 1.3 times higher chance of presenting with a CVD [78]. Thus, strategies directed towards controlling levels of IL-6 may be helpful in PLWH. This meta-analysis, found a significant change of lower levels of IL-6 after exercise intervention in the studies of Dudgeon et al. [58], where only males participated in an AERT intervention, and Zanetti et al. [60], where a mixed-gender intervention group performed only RT (see Table 4), supporting exercise as an alternative to reduce levels of IL-6 in PLWH.

No changes in IL-1β levels were found. This data is in agreement with Peake et al. [79], who reported that even though AE and RT increase muscle and leucocyte cells’ gene expression of IL-1β, the release of this interleukin is highly regulated and thus changes in plasma may not be observed.

## Limitations

The high degree of heterogeneity found in the meta-analyses was considered to be the result of a combination of various differences among the studies included in the quantitative analysis. Associated comorbidities in PLWH included coinfection of the hepatitis C virus [70,71], unhealthy habits like smoking, drinking or the use of hallucinogenic drugs [67,69,70] and other diagnosed chronic pathologies [57,67,69,71]. Gender, type of training, strength performance testing, active or passive control groups and the number of participants in each analysis was also uneven among the studies. After exploring some potential sources of heterogeneity, such as the exercise characteristics (exercise intensity, volume and weekly frequency). We were able to reduce the heterogeneity from high to low or from high to moderate in some cases. However, a high heterogeneity was still observed for most analyses. This heterogeneity can be attributed to the lack of power of the I^2^ statistic when looking to small number of studies. [80–82]. For these reasons, the results of this meta-analysis warrant attention when interpreted.

Other limitation was the presence of publications bias on the systematic review. According to the egger´s test, there ar publication bias for the meta-analyses: chest press (bias = 10.6, 95%CI: 6.3–14.9, p < 0.001), leg press (bias = 7, 95%CI: 2.7–11.4, p = 0.009) and leg flexion (bias = 7.4, 95%CI: 0.6–14.1, p = 0.03).

Regarding the clinical relevance of muscle strength in PLWH, the question remains, whether a value lower or higher than 40% could reflect a better cut-off point.

## Implications for research

After the above studies were evaluated in this meta-analysis, some concerns, pertinent for further RCTs involving exercise interventions in PLWH were raised. From a methodological standpoint, when possible, equally balanced groups in terms of gender, use of a unique type of training and blinding of evaluators could all increase the quality of future RCTs and decrease the risk of bias. Additionally, more studies investigating changes in testosterone and inflammatory markers (IL-6 and IL-1β) to establish the effect of RT alone or AERT on hormones in PLWH are needed.

## Conclusion

Resistance training alone or combined with aerobic exercise showed a positive change after studies with a low risk of bias and professional supervision were analyzed, improving upper and, more critically, lower body muscle strength. This meta-analysis also reported that exercise had a lowering effect on IL-6 levels in PLWH.

## Key considerations

- Both RT alone and AERT have a significant positive effect on increasing upper and lower body muscle strength in PLWH.
- Lower body strength increases more if exercise is supervised by a professional.
- Exercise is an alternative to reduce levels of IL-6 in PLWH.
- Taken together, exercise plays a fundamental role in HIV treatment to improve upper and lower body muscle strength in PLWH.

## Supporting information

S1 Table Information on excluded and included studies. (XLS)

S1 PRISMA Checklist (DOC)

## Abbreviations

95% CI: 95% Confidence interval
AE: Aerobic exercise
AERT: Aerobic exercise combined with resistance training
AH: Andreas Heissel
BMD: Bone mineral density
CP: Camilo Pérez-Chaparro
ART: Combined anti-retroviral drug therapy
MeSH: Combined Medical Subject Headings
CG(s): Control Group(s)
EC: Eligibility Criteria
I^2^: Heterogeneity
HIV: Human immunodeficiency virus
IL-1β: Interleukin 1β
IL-6: Interleukin 6
IQR: Interquartile range
IG(s): Intervention group(s)
kg: Kilograms
HR_max_: Maximum heart rate
μg/ml: Micrograms per milliliter
mmol/l: Millimoles per liter
cells·ml^-1^: Number of cells per microliter
n: Number of studies/participants
PLWH: People living with HIV
PZ: Philipp Zech
pg/dl: Picograms per deciliter
lbs: Pounds
PRISMA: Preferred Reporting Items for Systematic Reviews and Meta-Analyses
RCTs: Randomized controlled trials
RT: Resistance training alone
SD: Standard error
WMD: Weighted mean difference
